# Direct hospitalization costs of children with *Mycoplasma pneumoniae* pneumonia from 2013 to 2025 in China: a longitudinal retrospective single-center study

**DOI:** 10.3389/fpubh.2026.1762614

**Published:** 2026-02-17

**Authors:** Anle Shen, Zhiling Li, Shiying Huang, Tao Xu, Yejian Wang, Yong Yin, Jiande Chen

**Affiliations:** 1 Department of Clinical Pharmacy, Shanghai Children's Medical Center, Shanghai Jiao Tong University School of Medicine, Shanghai, China; 2 Department of Pharmacy, Hainan Branch, Shanghai Children's Medical Center, School of Medicine, Shanghai Jiao Tong University, Sanya, China; 3 Department of Respiratory Medicine, Shanghai Children's Medical Center, Shanghai Jiao Tong University School of Medicine, Shanghai, China; 4 Department of Respiratory Medicine, Shanghai Children's Medical Center GuiZhou Hospital, Shanghai Jiao Tong University School of Medicine, Guiyang, Guizhou, China

**Keywords:** children, cost, hospitalization, *Mycoplasma pneumoniae*, pneumonia

## Abstract

**Background:**

*Mycoplasma pneumoniae* pneumonia (MPP) is one of the most common type of community-acquired pneumonia. Due to the diversity in treatment measures and macrolide-resistant, the medical burden of hospitalized children remains uncertain.

**Methods:**

This study conducted a retrospective analysis of pediatric patients diagnosed with MPP and hospitalized at Shanghai Children's Medical Center over a 13-year period, from January 2013 to June 2025. The duration of hospital stay, total hospitalization costs, and drug costs per hospitalization were analyzed by year, corticosteroid administration, and presence of macrolide-resistant genes.

**Results:**

A total of 4,684 hospitalized children with MPP were included. The median age of the cohort was 6.50 years (quartile: 4.20–8.30 years), and the median duration of hospital stay was 5.00 days (quartile: 4.00–7.00 days). The median cost per hospitalization was $1,250.52 (quartile: $1,016.06–$1,612.27), while the median drug cost per hospitalization was $124.02 (quartile: $75.00–$199.31). Significant differences were observed in the duration of hospital stay, total costs, and drug costs per hospitalization between the age groups < 3, 3–6, 6–10, and ≥10 years. Additionally, significant differences were found between patients who received corticosteroids and those who did not, in terms of hospital stay duration, total costs, and drug costs per hospitalization.

**Conclusions:**

The medical burden associated with MPP in children under 3 years of age warrants attention and should be a focus of medical reimbursement policy considerations.

## 1 Introduction

*Mycoplasma pneumoniae* (MP), an atypical bacterium, is a prominent pathogen responsible for respiratory infections in children, contributing to up to 40% of community-acquired pneumonia (CAP) cases in this demographic globally (1, 2). In contrast to viral or typical bacterial pathogens, MP demonstrates distinctive epidemiological patterns, including cyclical epidemics occurring every 4–7 years and predilection for school-aged children (3). Although MP pneumonia (MPP) is generally self-limiting and rarely results in direct mortality (< 0.1%), its involvement in precipitating severe complications indirectly contributes to pneumonia-related fatalities (3, 4). The ongoing emergence of macrolide-resistant MP (MRMP), particularly due to mutations at positions 2063 (A2063G/C) and 2064 (A2064G/C) in the V domain of the 23S rRNA of MP, is associated with more severe pneumonia and a range of extrapulmonary complications, including pleuritis, pericarditis, and encephalitis (1, 3).

Due to the absence of a cell wall in MP, macrolides, such as azithromycin, clarithromycin and erythromycin, are considered the most effective and primary therapeutic agents for treating macrolide-susceptible or mild MPP (3, 5, 6). In cases of pneumonia induced by MRMP, newer tetracycline antibiotics, including doxycycline and minocycline, as well as fluoroquinolones, such as levofloxacin and moxifloxacin, are recommended as alternative treatments (3, 5, 7). Corticosteroids are advised for managing MPP with pronounced inflammatory responses (3). Beyond pharmacological interventions, the management of MPP necessitates radiological and laboratory assessments, bronchoscopy-guided examinations, interventional therapies, and adjunctive treatments, such as oxygen supplementation for hypoxia and humidified air (1, 5, 6). Consequently, the economic burden associated with MPP is significant and should not be overlooked. While there is existing research on the economic impact of CAP and infections caused by *pneumococcal* or *respiratory syncytial virus* (RSV) (8–11), there is a notable lack of studies addressing the direct medical costs of MPP in pediatric populations.

This study sought to quantify the direct hospitalization costs associated with pediatric MPP in China, with the aim of enhancing the efficiency of medical resource utilization, helping evaluate future prevention efforts and providing a foundation for government departments to formulate evidence-based health policies and public health intervention strategies.

## 2 Materials and methods

### 2.1 Analytics overview

This retrospective study conducted an analysis of patient data from Shanghai Children's Medical Center (SCMC) over a 13-year period, spanning from January 2013 to June 2025. The inclusion criteria encompassed children with a primary diagnosis of MPP, as well as hospitalizations where CAP was the primary diagnosis and MPP was a secondary diagnosis. The exclusion criteria included patients over 18 years of age, children who were immunocompromised due to antineoplastic or immunosuppressive therapy, those admitted to intensive care units, and transplant recipients. The cohort was stratified into four age groups based on previous literatures: < 3 years (infants and toddlers), 3–6 years (preschool), 6–10 years (school-age) and ≥10 years (adolescent) (12, 13).

Data were extracted from the SCMC Hospital Information System, capturing variables such as patient age, duration of hospital stay, hospitalization costs, and drug costs per hospitalization. Macrolide-resistant gene testing was initiated in January 2020. Mutations at positions 2063 and 2064 in the V domain of the 23S rRNA of MP were identified through direct sequencing of samples that yielded positive results in polymerase chain reaction assays (14). Subsequently, data relevant to this testing were collected from that point onward. All patient data were anonymized throughout the study. The following exchange rate was used to convert Chinese Yuan into US dollars in this study: 1 US$ = 7.20 CNY (July 2025).

The study was conducted in accordance with the Declaration of Helsinki, and the protocol was approved by the Shanghai Children's Medical Center Ethics Committee (No. SCMCIRB-YPDKJW2023002, 21 February 2023). Informed consent was waived due to the retrospective nature of this study.

### 2.2 Analyses

Data analysis was conducted using IBM SPSS, version 25.0. The Kolmogorov-Smirnov test was utilized to assess the normality of the data distribution. Continuous variables following a normal distribution were reported as means (±standard deviation), while those not normally distributed were presented as medians (interquartile range). Categorical variables were expressed as frequencies (percentages). Significant differences between two groups with non-normal distributions were assessed using the Mann-Whitney *U* test, whereas the Kruskal-Wallis test was employed for comparisons among three or more groups with non-normal distributions. As a *post hoc* test, the Dunn test with Bonferroni correction was used following the Kruskal-Wallis tests. Statistical significance was determined at a threshold of *P* < 0.05 (two-tailed).

## 3 Results

### 3.1 Characteristics of study subjects

A total of 4,684 hospital admissions were included in this study, with a male-to-female ratio of 49:51. The < 3 years group comprised 625 patients (13.34%), the 3–6 years group included 1,360 patients (29.04%), the 6–10 years group included 2,108 patients (45.00%), and the ≥10 years group consisted of 591 patients (12.62%). Corticosteroids were administered to 3,599 patients (76.84%). Macrolide-resistant gene testing was conducted on 2,148 patients, revealing that 1,759 (81.89%) had MRMP pneumonia (MRMPP; Table 1).

**Table 1:** Patient characteristics.

**Characteristic**	***n* (%)**
Male	2,296 (49.02)
Female	2,388 (50.98)
< 3 years	625 (13.34)
3–6 years	1,360 (29.04)
6–10 years	2,108 (45.00)
≥10 years	591 (12.62)
Corticosteroids administrated	3,599 (76.84)
Macrolide-resistant gene detected^a^	1,759 (81.89)

^a^Macrolide-resistant gene testing was implemented in January 2020, with 2,148 patients were given macrolide-resistant gene testing.

### 3.2 Direct hospitalization costs by year

The median age of patients hospitalized was 6.50 years (quartile: 4.20–8.30 years). The median duration of hospital stay was 5.00 days (quartile: 4.00–7.00 days). The median total cost per hospitalization amounted to $1,250.52 (quartile: $1,016.06–$1,612.27), while the median drug cost per hospitalization was $124.02 (quartile: $75.00–$199.31). The number of hospitalizations was 643 in 2019, and increased significantly in 2023 and 2024, which were 1,394 and 1,766, respectively. The annual median ages of hospitalized patients from 2013 to 2025 ranged from 5.35 to 8.15 years. The annual median duration of hospital stays ranged from 5.00 to 7.57 days. The annual median overall cost per hospitalization peaked in 2022 at $1,415.18 and was lowest in 2015 at $785.97. The annual median drug cost per hospitalization was highest in 2013 at $345.22 and lowest in 2024 at $94.79 (Table 2).

**Table 2:** Direct hospitalization costs of children with *Mycoplasma pneumoniae* pneumonia^a^.

**Year**	**No. of hospitalizations**	**Median age**	**Median duration of hospital stay (days)**	**Median total cost per hospitalization ($)**	**Median drug cost per hospitalization ($)**
2013	21	6.25 (±2.62)^b^	7.57 (±2.16)^b^	999.88 (±399.81)^b^	345.22 (±150.38)^b^
2014	13	7.17 (±2.76)^b^	6.00 (5.00–7.00)	999.80 (±270.19)^b^	235.50 (±101.31)^b^
2015	25	5.80 (±2.74)^b^	6.00 (5.00–8.00)	785.97 (637.03–1,241.33)	200.07 (119.56–413.45)
2016	29	6.90 (6.15–8.70)	7.24 (±2.92)^b^	1,036.16 (818.10–1,287.24)	232.25 (133.00–291.46)
2017	29	7.10 (±2.12)^b^	5.00 (4.00–6.00)	949.18 (746.15–1,231.80)	208.89 (140.87–268.66)
2018	21	6.46 (±1.99)^b^	5.00 (4.00–6.00)	1,088.56 (927.84–1,291.68)	147.54 (133.11–256.96)
2019	643	5.60 (3.50–7.40)	6.00 (4.00–7.00)	1,156.80 (955.72–1,447.98)	188.45 (129.22–257.14)
2020	140	5.35 (3.20–7.68)	7.00 (5.00–8.00)	1,172.88 (989.48–1,443.39)	196.74 (136.31–258.73)
2021	222	5.85 (3.70–7.70)	6.00 (5.00–8.00)	1,352.40 (1,145.76–1,783.20)	157.91 (102.61–246.82)
2022	225	6.00 (3.60–8.45)	6.00 (5.00–7.00)	1,415.18 (1,205.40–1,734.45)	130.05 (90.65–204.92)
2023	1,394	6.70 (4.70–8.60)	5.00 (4.00–6.00)	1,287.16 (1,070.89–1,688.65)	117.74 (74.49–199.29)
2024	1,766	6.60 (4.30–8.50)	5.00 (4.00–6.00)	1,256.67 (1,022.86–1,658.01)	94.79 (62.19–154.89)
2025	156	8.15 (5.43–10.18)	5.00 (4.00–6.00)	1,086.18 (891.73–1,580.72)	95.92 (64.97–153.07)
Total	4,684	6.50 (4.20–8.30)	5.00 (4.00–7.00)	1,250.52 (1,016.06–1,612.27)	124.02 (75.00–199.31)

^a^Variables with non-normal distribution were presented medians (interquartile range).

^b^Data normally distributed were presented as means (±standard deviation).

### 3.3 Comparisons grouped by age

The median duration of hospital stay for the < 3 years age group was 6 days, which was significantly different from the durations observed in the 3–6, 6–10 and ≥10 years age groups. The median total cost per hospitalization for the < 3, 3–6, 6–10 and ≥10 years age groups were $1,320.74, $1,273.32, $1,226.08 and $1,215.24, respectively. Pairwise comparisons revealed significant differences between the < 3 years group and both the 6–10 and ≥10 years groups. The median drug cost per hospitalization for the < 3 and 3–6 years group were $132.19 and $132.61, respectively, which were significantly higher than those for the 6–10 and ≥10 years age groups (Table 3).

**Table 3:** Comparisons of the <3 years group, the 3–6 years group, the 6–10 years group and the ≥10 years group in median duration of hospital stay, median cost per hospitalization and median drug cost per hospitalization.

**Variables**	** < 3 years**	**3–6 years**	**6–10 years**	**≥10 years**	Kruskal-Wallis	
					***H***	***P*-value**
Median duration of hospital stay (days)^a^	6.00 (5.00–7.00)	5.00 (4.00–7.00)	5.00 (4.00–6.00)	5.00 (3.00–6.00)	170.53	< 0.001
Median total cost per hospitalization ($)^b^	1,320.74 (1,079.10–1,566.04)	1,273.32 (1,041.77–1,641.59)	1,226.08 (993.26–1,619.87)	1,215.24 (987.07–1,613.80)	15.31	0.002
Median drug cost per hospitalization ($)^c^	132.19 (91.54–204.55)	132.61 (82.08–210.98)	118.53 (71.30–196.51)	108.97 (64.66–179.38)	55.75	< 0.001

^a^Kruskal-Wallis test revealed significant differences between all group pairs.

^b^Kruskal-Wallis test showed that significant differences were observed between the < 3 years group and both the 6–10 and ≥10 years groups.

^c^Kruskal-Wallis test showed that significant differences were found between all group pairs except between the < 3 and 3–6 years groups.

### 3.4 Comparisons grouped by with/without corticosteroids administration

Significant differences were observed between the no corticosteroids group and the corticosteroids group in terms of median duration of hospital stay, median total cost per hospitalization, and median drug cost per hospitalization. Both groups had a median hospital stay duration of 5 days. However, the median total cost per hospitalization was higher in the corticosteroids group ($1,287.74) compared to the no corticosteroids group ($1,157.64). Additionally, the median drug cost per hospitalization was significantly greater in the corticosteroids group, amounting to $129.16 (Table 4).

**Table 4:** Comparisons of the no corticosteroids group and the corticosteroids group in median duration of hospital stay, median cost per hospitalization and median drug cost per hospitalization.

**Variables**	**No corticosteroids**	**Corticosteroids**	***Z***	***P*-value**
Median duration of hospital stay (days)	5.00 (4.00–6.00)	5.00 (4.00–7.00)	9.16	< 0.001
Median total cost per hospitalization ($)	1,157.64 (949.80–1,376.26)	1,287.74 (1,049.40–1,698.57)	10.92	< 0.001
Median drug cost per hospitalization ($)	113.11 (73.54–169.15)	129.16 (75.63–209.63)	5.67	< 0.001

### 3.5 Comparisons grouped by with/without macrolide-resistant gene

Significant differences were observed in the median duration of hospital stay between the group without macrolide-resistant genes (5 days) and the group with macrolide-resistant genes (5 days). However, no significant differences were identified in the median total cost per hospitalization or the median drug cost per hospitalization. Specifically, the median total cost per hospitalization for the group without macrolide-resistant genes was $1,370.66, compared to $1,400.39 for the group with macrolide-resistant genes. Similarly, the median drug cost per hospitalization was $121.26 for the former group and $131.91 for the latter (Table 5).

**Table 5:** Comparisons of the no macrolide-resistant gene group and the macrolide-resistant gene group in median duration of hospital stay, median cost per hospitalization and median drug cost per hospitalization.

**Variables**	**No macrolide-resistant gene**	**Macrolide-resistant gene**	***Z***	***P*-value**
Median duration of hospital stay (days)	5.00 (4.00–6.00)	5.00 (4.00–7.00)	4.29	< 0.001
Median total cost per hospitalization ($)	1,370.66 (1,169.23–1,745.29)	1,400.39 (1,192.58–1,855.40)	1.69	0.09
Median drug cost per hospitalization ($)	121.26 (85.03–195.07)	131.91 (88.61–200.50)	1.35	0.18

## 4 Discussion

With ongoing advancements in disease diagnosis and treatment methodologies, there is an increasing scholarly emphasis on the treatment burden associated with various diseases (8, 15). The pursuit of more effective therapeutic strategies at reduced costs has emerged as a central research priority. Nonetheless, research specifically addressing the economic aspects of MPP in pediatric populations remains notably limited. To address this gap, this retrospective study analyzed the direct medical costs associated with pediatric MPP hospitalizations at SCMC over a 13-year period. Our findings offer valuable insights for optimizing clinical decision-making, enhancing the efficiency of healthcare resource utilization, and alleviating the financial burden on affected families. Furthermore, this study provides quantitative evidence to inform governmental health resource allocation priorities and the development of policies aimed at mitigating the total disease burden.

Our study determined that the median age of patients with MPP was 6.50 years, aligning with the established epidemiological characteristics of MPP (3). The duration of hospital stay ranged from 4.00 to 7.00 days, which was in line with that reported in a prior Chinese study (16), suggesting that the condition of MPP typically improves after approximately 1 week of hospitalization. Furthermore, the median total cost per hospitalization in this study amounted to $1,250.52, surpassing the direct hospitalization cost of $851.48 reported in the prior study conducted in Shandong, which did not account for medical insurance reimbursement (16). This discrepancy is largely due to the variation in medical service prices across different provinces and cities. These prices are influenced by factors such as the level of local economic development, per capita income, public payment capacity, and the actual operational costs incurred by medical institutions (17). Additionally, pharmaceutical expenses constituted approximately 10% of the total direct hospitalization costs, indicating that controlling non-pharmaceutical expenditures, such as those related to radiological and laboratory examinations, is crucial for managing overall hospitalization costs.

Our research identified a notable rise in the incidence of MPP during the 2023–2024 period, which may be associated with the implementation of non-pharmaceutical interventions (NPIs) designed to curb the spread of COVID-19, a situation described as “immunity debt” (4, 18). The NPIs disrupted the transmission dynamics of MP, resulting in inadequate immune system stimulation, a reduction in herd immunity, and an increased vulnerability to infection. Following the relaxation of NPIs in late 2022, there was a widespread outbreak of SARS-CoV-2, which led to prolonged immune suppression or dysregulation, especially in children, thereby elevating their susceptibility to MPP (4). This post-COVID epidemiological transition has consequently contributed to an increase in MPP hospitalizations and severity within pediatric populations.

Interestingly, there has been a notable decline in both the total cost per hospitalization and the drug cost per hospitalization over the past 5 years, a trend likely attributable to the national volume-based procurement policies initiated in December 2018 (19). These policies were designed to ensure the provision of high-quality and affordable pharmaceuticals and medical supplies by establishing a national drug public procurement market and a multi-party linkage procurement model (20). The national volume-based procurement policies mandated specific purchase volumes, required an upfront payment of 30%-50%, and employed a competitive bidding process. As a result, the average reduction in drug prices has exceeded 50%, leading to significant savings in drug expenditures (19, 21). Consequently, it is crucial to further refine these volume-based pharmaceutical procurement policies to effectively manage hospitalization costs.

Inflammatory response is a significant characteristic of MPP, particularly in pediatric cases of MRMPP and severe MPP (22–24). It is recommended that MPP cases exhibiting elevated levels of white blood cells, neutrophil percentage, C-reactive protein, procalcitonin, interferon-γ, and interleukin-6 be treated with corticosteroids (3). In our study, approximately three-quarters of hospitalized children received corticosteroid treatment. The findings indicated significant differences in the duration of hospital stay, total cost per hospitalization, and drug cost per hospitalization between the corticosteroid-treated group and the non-corticosteroid group. Due to the retrospective nature of this study, accurately distinguishing between severe and general MPP was not feasible. Previous research has demonstrated that the severe MPP group exhibits significantly higher levels of inflammatory cytokines compared to the general MPP group (24). It is plausible that the MPP cases in the corticosteroid group were more severe than those in the non-corticosteroid group, necessitating more intensive interventions, such as bronchoscopy-guided examination, which contributed to prolonged hospital stays and increased hospitalization costs.

As indicated in previous studies (25, 26), the detection rate of MRMP in this study was 81.89%. The findings demonstrated no significant differences in total cost or drug expenses per hospitalization between the groups with and without macrolide-resistant genes. The administration of newer tetracycline antibiotics or other effective drugs to children with MRMPP, upon detection of the macrolide-resistant gene, could significantly alleviate pneumonia symptoms and facilitate early recovery (27). Currently, pathogen diagnostic technologies are advancing rapidly. A variety of diagnostic methods, including molecular diagnostic techniques, targeted next-generation sequencing, and polymerase chain reaction for MP, have enhanced the capacity to detect MPP or MRMPP in children (6). Notably, targeted next-generation sequencing, which allows for the simultaneous and accurate identification of multiple pathogens, is increasingly being adopted (28). The outcomes of this study may support the implementation of rapid molecular diagnostics for precise pathogen targeting.

Our study also found that the hospitalization total cost was highest among children under 3 years of age, potentially due to the increased likelihood of mixed viral infections within this age group (29). Children with MPP who also have infections with viruses such as RSV, *human rhinovirus, adenovirus*, and *human metapneumovirus* may experience prolonged hospital stays and impose additional financial burdens on healthcare systems (29). To effectively manage medical expenses, the Diagnosis-Related Group (DRG) payment system has been implemented as a key cost-containment strategy in numerous countries worldwide (30–32). Based on our findings, it is recommended that the National Healthcare Security Administration consider age-based grouping when formulating DRG policies related to MPP. Specifically, the group of children under 3 years of age with MPP should receive increased medical reimbursement.

Nonetheless, there were some limitations of this study. Firstly, the research was conducted at a single hospital located in Shanghai, a region characterized by relatively rapid economic development in eastern China, which may introduce potential biases. Our research team intends to continuously monitor the medical burden of MPP in future studies to enhance the outcomes. Secondly, the study was unable to differentiate between severe and general MPP due to incomplete data, resulting in the inability to ascertain the hospitalization costs for children with severe MPP. Future prospective studies will be conducted to assess the medical burden of both severe and general MPP, thereby providing a foundation for refining DRG policies. Thirdly, a limitation of this 13-year retrospective study was the potential for inconsistent documentation and missing data, which hindered the identification of factors influencing the medical burden. To address these challenges, future prospective research should aim to accurately ascertain the determinants of medical burden. Fourthly, owing to technical constraints and challenges in data accessibility, the cost data spanning the 13-year period in this study utilized a consistent exchange rate, and adjustments for inflation were not incorporated. This methodological choice may impact the temporal comparability of the economic data.

In conclusion, the medical burden of MPP has remained relatively stable. It is recommended that children under the age of three with MPP be allocated higher medical reimbursement expenditures. Additionally, the immediate administration of susceptible medications is advised upon detection of the macrolide-resistant gene.

## Data Availability

The original contributions presented in the study are included in the article/supplementary material, further inquiries can be directed to the corresponding authors.
